# Demystifying the power of essential oils: a review of their antibacterial properties and potential as natural food preservatives

**DOI:** 10.17179/excli2025-8439

**Published:** 2025-07-17

**Authors:** Hanen Falleh

**Affiliations:** 1 Laboratory of Aromatic and Medicinal Plant, Center of Biotechnology of Borj Cedria, BP 901, Hammam-Lif 2050, Tunisia

**Keywords:** Essential oils, Antimicrobial mechanisms, Foodborne pathogens, Natural food preservatives, Encapsulation techniques

## Abstract

This review delves into the antimicrobial potential of essential oils (EOs), focusing on their mechanisms of action against foodborne pathogens and their applications as natural food preservatives. EOs, derived from aromatic plants, are complex mixtures of volatile compounds, primarily terpenes, terpenoids, and phenolic compounds, which exhibit potent antimicrobial properties. These bioactive compounds disrupt bacterial cell membranes, increase permeability, and induce leakage of intracellular components, leading to cell death. Additionally, EOs inhibit energy production by depleting ATP levels, disrupting the tricarboxylic acid cycle, and impairing cellular respiration. They also generate reactive oxygen species (ROS), causing oxidative stress and damaging bacterial DNA, proteins, and lipids. Furthermore, EOs interfere with quorum sensing and biofilm formation, reducing bacterial virulence and resistance. Despite their efficacy, challenges such as strong flavors, poor solubility, and environmental sensitivity limit their direct application. To address these issues, encapsulation techniques, such as nanoemulsions and active packaging, have been developed to enhance the stability and controlled release of EOs. For instance, nanoencapsulation of thyme and cinnamon EOs has significantly improved their antimicrobial efficacy in food products like milk and minced meat. By harnessing the multifaceted mechanisms of EOs, this review underscores their potential as sustainable and effective natural preservatives to combat foodborne pathogens and improve food safety.

See also the graphical abstract(Fig. 1).

## 1. Introduction

Food safety continues to be one of the most critical global challenges, as foodborne illnesses remain a major cause of morbidity and mortality worldwide. Current estimates from the World Health Organization (2022) reveal a staggering global health burden from food contamination, with epidemiological data showing approximately 600 million annual cases of foodborne illness (equivalent to one-tenth of humanity) and 420,000 subsequent deaths that proper food handling could prevent. (WHO, 2022[64]). These diseases are primarily caused by pathogenic microorganisms such as *Salmonella* spp., *Escherichia coli*, *Listeria monocytogenes*, and *Campylobacter* spp., which can contaminate food at various stages of the supply chain (EFSA, 2024[24]).

To mitigate these risks, the food industry has traditionally relied on synthetic preservatives such as benzoates, nitrates, sorbates, and sulfites to inhibit microbial growth and prolong shelf life. However, increasing evidence has raised concerns regarding the long-term consumption of these additives. Several studies have linked synthetic preservatives to adverse effects including hypersensitivity reactions, carcinogenicity, and interference with gut microbiota (Ahmed et al., 2021[1]; Chaichi et al., 2021[12]; Radunz et al., 2020[55]; Jayari et al., 2018[32]). Furthermore, there is a growing consumer demand for natural, minimally processed, and "clean label" foods, which has led to an intensified search for effective natural preservatives (Falleh et al., 2020[27]).

Among the various natural alternatives, essential oils (EOs) have gained considerable attention due to their potent antimicrobial properties and natural origin. Essential oils are volatile, aromatic compounds extracted from different parts of plants such as flowers, leaves, bark, seeds, or roots. They are composed of complex mixtures of terpenes, terpenoids, phenols, aldehydes, ketones, and alcohols, many of which have demonstrated antimicrobial, antioxidant, anti-inflammatory, and antifungal effects (Souiy, 2024[60]; Sadgrove et al., 2022[59]). Traditionally used in perfumery and medicine, essential oils are now being investigated for their efficacy in food preservation and pathogen control.

The growing interest in essential oils as antibacterial agents is fueled by their broad-spectrum activity against a variety of foodborne pathogens. Numerous studies have confirmed the effectiveness of EOs from thyme, oregano, clove, cinnamon, and tea tree, among others, against pathogens such as *E. coli* O157:H7, *Listeria monocytogenes*, *Campylobacter jejuni*, and *Salmonella enterica* (Yuan et al., 2019[66]; Lin et al., 2019[44]; Kazemeini et al., 2019[37]; Jayari et al., 2018[32]). Their modes of action are multi-faceted and include disruption of microbial membranes, leakage of intracellular contents, inhibition of enzyme activity, and suppression of quorum sensing, which is essential for microbial communication and biofilm formation (Mehidi et al., 2024[46]).

One of the advantages of essential oils is their ability to target multiple cellular mechanisms, making it more difficult for bacteria to develop resistance compared to single-target antibiotics. This characteristic makes EOs particularly relevant in the era of antibiotic resistance, where multidrug-resistant strains of foodborne pathogens are becoming increasingly common (Chen et al., 2023[14]; Qian et al., 2019[54]). Furthermore, essential oils often exhibit synergistic effects when combined with conventional antimicrobials or other natural compounds, allowing for lower effective doses and improved safety profiles (Chaichi et al., 2021[12]). In addition to their antimicrobial potential, essential oils have demonstrated a wide range of direct health benefits for humans. These include antioxidative protection against oxidative stress, anti-inflammatory properties that may help manage chronic inflammatory conditions, and anxiolytic or antidepressant effects that support mental well-being (Figure 2(Fig. 2)). Some EOs have also shown anticancer and hepatoprotective activities, underscoring their therapeutic promise. The holistic bioactivity profile of essential oils makes them attractive candidates not only for food preservation but also for nutraceutical and medicinal applications (Burt, 2004; Sharifi-Rad et al., 2017).

Despite their promising antimicrobial potential, the direct application of essential oils in food products presents several challenges. Their strong aroma and flavor may negatively affect the sensory attributes of food, particularly when high concentrations are required for microbial inhibition. Additionally, their volatility, poor water solubility, and sensitivity to environmental factors such as temperature, oxygen, and light can limit their effectiveness in food matrices (Ben Jemaa et al., 2018[6]). To overcome these limitations, various strategies have been developed to enhance the delivery and functionality of essential oils in food systems. Encapsulation technologies, such as nanoemulsions, liposomes, cyclodextrins, and biopolymeric coatings, have been widely investigated to protect essential oils from degradation, improve solubility, and allow for controlled release (Casalini and Giacinti Baschetti, 2023[11]; Dghais et al., 2023[18]; Falleh et al., 2021[26]; Hassoun & Çoban, 2017[29]). Nanoencapsulation, in particular, has shown promise in improving the antibacterial efficacy of EOs against resistant strains, while minimizing sensory impact on food products (Ben Jemaa et al., 2018[7]).

Recent research has also explored the incorporation of essential oils into active packaging materials, enabling continuous release of antimicrobial compounds during storage. Such systems not only improve shelf life but also reduce the need for direct addition of preservatives to food, thus meeting the growing demand for clean-label solutions. Moreover, combinations of essential oils with other natural antimicrobials-such as nisin, lysozyme, or plant phenolics-have demonstrated synergistic effects, providing enhanced microbial control with lower concentrations of each agent (Lin et al., 2019[44]; Blanco-Lizarazo et al., 2017[8]; Khaleque et al., 2016[38]).

From a regulatory standpoint, several essential oils and their active constituents are classified as Generally Recognized As Safe (GRAS) by the U.S (Anis et al., 2020[5]). Food and Drug Administration, which has further encouraged their application in the food industry. Nevertheless, the regulatory landscape is still evolving, and comprehensive toxicological evaluations are necessary to ensure consumer safety, especially for long-term or high-dose applications.

As the food industry continues to seek sustainable, natural, and effective solutions to microbial contamination, essential oils stand out as promising candidates. Their multifunctional bioactivity, coupled with consumer preference for natural ingredients, positions them as key tools in the development of next-generation food safety technologies.

## 2. Essential Oils Presentation

### 2.1. Overview of EOs 

Essential oils (EOs) are highly concentrated, hydrophobic liquids containing volatile chemical constituents derived from aromatic plants. They are typically extracted through processes such as hydrodistillation, steam distillation or cold pressing, with the specific method significantly influencing their composition and quality (Souiy, 2024[60]). These volatile organic compounds are primarily composed of phenolic compounds (phenylpropanoids) and isoprenoids, including monoterpenes and sesquiterpenes, which together contribute to their distinctive biological activities (Falleh et al., 2020[27]). EOs are synthesized by plants as secondary metabolites, serving diverse functions. The primary function of essential oils is to provide scent and flavour to plants. They also play an important communicative role as deterring herbivores, attracting pollinators, and providing defense against microbial pathogens. Sometimes, they also act as signals for other plants of the same species (Durczyńska and Żukowska, 2024[22]). They can be derived from various parts of plants, including leaves, flowers, bark, seeds, and roots. Due to their complex chemical composition, essential oils exhibit a wide array of bioactivities, including antimicrobial, antioxidant, anti-inflammatory, and antifungal properties, making them invaluable across multiple industries such as food preservation (Pezantes-Orellana et al., 2024[53]). To provide a deeper understanding of essential oils, table 1(Tab. 1) (References in Table 1: Falleh et al., 2020[27]; Kuttan and Liju, 2017[42]; Meryani et al., 2024[47]; Naja et al., 2022[49]; Pezantes-Orellana et al., 2024[53]; Sadgrove et al., 2022[59]; Souiy, 2024[60]) summarizes key aspects such as their chemical composition, biosynthesis, biological roles, and applications.

### 2.2. Essential oils chemical composition

Major bioactive constituents of EOs include terpenes, terpenoids and phenolic compounds (Table 2(Tab. 2)) that often act synergistically, enhancing the effectiveness of essential oils in combating oxidative stress and pathogenic microorganisms (Sadgrove et al., 2022[59]). The chemical composition of essential oils can be broadly categorized into the following key classes of molecules (Sadgrove et al., 2022[59], Falleh et al., 2020[27]):

#### Terpenes

Terpenes are the most abundant class of compounds in essential oils, synthesized *via* the mevalonate (MVA) and methylerythritol phosphate (MEP) pathways from isoprene (C5) units. They are hydrocarbons, but some undergo oxidation to form oxygenated derivatives.

*Monoterpenes* (C10): These compounds, composed of two isoprene units, include limonene, pinene, and myrcene. They are highly volatile, responsible for the fresh and citrusy aroma in many essential oils and often exhibit antimicrobial and anti-inflammatory properties.

*Sesquiterpenes* (C15): Formed from three isoprene units, they are less volatile than monoterpenes and contribute to the oil's depth and longevity. Examples include *β*-caryophyllene and humulene, which have anti-inflammatory and analgesic properties.

*Diterpenes* (C20): These are composed of four isoprene units and are less common in essential oils due to their high molecular weight and low volatility. Some diterpenes, such as sclareol in clary sage, exhibit antimicrobial and anticancer properties.

#### Oxygenated Compounds

These compounds result from the oxidation of hydrocarbons and enhance both the aromatic complexity and biological activity of essential oils (Sadgrove et al., 2022[59]).

*Alcohols*: Contain a hydroxyl (-OH) functional group. Examples like linalool (C_10_H_18_O) and geraniol (C_10_H_18_O) are known for their floral and sweet aromas, as well as antimicrobial and calming effects.

*Aldehydes*: Characterized by a carbonyl (-CHO) group, these compounds, such as cinnamaldehyde (C_9_H_8_O) from cinnamon, contribute spicy, warm scents and exhibit strong antibacterial and antifungal activity.

*Ketones*: Contain a carbonyl (C=O) group within the molecule. Examples like camphor (C_10_H_16_O) are known for their penetrating aroma and therapeutic effects on respiration and circulation.

*Esters:* Formed from alcohols and acids, these compounds, such as linalyl acetate (C_12_H_20_O_2_) in lavender oil, contribute fruity and floral notes and often have sedative and anti-inflammatory properties.

*Phenols:* Aromatic compounds with a hydroxyl (-OH) group attached to a benzene ring. Eugenol (C_10_H_12_O_2_), found in clove oil, is known for its antiseptic and antioxidant properties.

*Oxides:* These are oxygenated terpenes, often formed by the oxidation of sesquiterpenes. An example is caryophyllene oxide (C_15_H_24_O), which contributes to woody, spicy aromas and has bioactive properties.

#### Phenylpropanoids

Derived from the shikimate pathway, phenylpropanoids are structurally distinct from terpenes, containing an aromatic benzene ring with a three-carbon (C_3_) propene tail. Examples include Eugenol (C_10_H_12_O_2_): A key component of clove oil, known for strong antiseptic and analgesic properties. Anethole (C_10_H_12_O): Found in anise and fennel, giving a sweet, licorice-like aroma with antispasmodic and antimicrobial benefits.

#### Other Compounds

Essential oils may contain trace amounts of additional compounds that modify aroma, enhance stability, or contribute bioactivity: Straight-chain aldehydes (e.g., n-decanal), contributing to citrusy or soapy scents. Indole (C_8_H_7_N): Found in jasmine and neroli, contributing floral and musky notes. Anthranilic acid esters: Present in some floral oils, adding sweet and musky undertones.

## 3. Essential Oils Mechanism of Action as Natural Antibacterial

**Figure 1:**
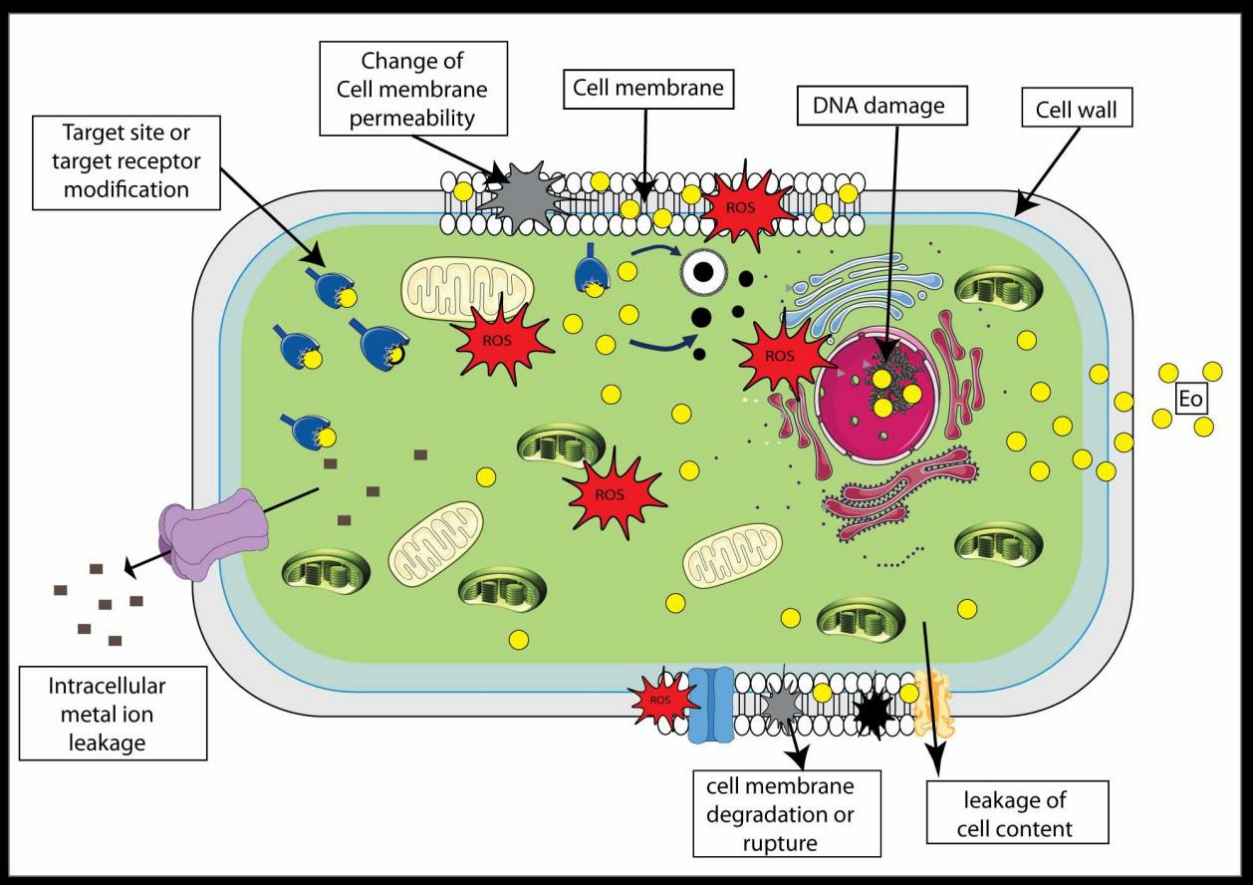
Graphical abstract: Main antimicrobial mechanisms of essential oils (EOs), including membrane disruption, increased permeability, ROS generation, DNA damage, and leakage of intracellular contents.

The exact mechanisms behind the antibacterial effects of essential oils are not yet fully understood. This complexity stems from the unique chemical composition and distinct biological properties of each essential oil, coupled with the varying sensitivities of different microorganisms (Anis et al., 2020[5]). Consequently, proposing a universal mechanism of action for all essential oils across all bacterial species is exceedingly challenging. Despite these complexities, numerous studies have shed light on the diverse mechanisms through which essential oils exert their antibacterial effects. These mechanisms often involve targeting multiple sites within bacterial cells, disrupting key physiological processes and structural integrity (Mehidi et al., 2024[46]). In some instances, the antimicrobial activity of essential oils cannot be linked to a single mode of action but rather results from a combination of biochemical and structural interactions occurring at various cellular sites, including the cell membrane and cytoplasm (Yap et al. 2021[65]). Below and in Figure 1(Fig. 1) (graphical abstract), we explore the primary modes of action, highlighting the multifaceted ways essential oils combat bacterial pathogens.

### Membrane Disruption and Increased Permeability

Essential oils (EOs) exert antibacterial effects primarily by targeting the bacterial membrane, leading to structural disruption and increased permeability. Their hydrophobic nature enables them to interact with membrane lipids, causing destabilization, leakage of intracellular components, and impairment of vital cellular functions. Both Gram-positive and Gram-negative bacteria are affected, though Gram-negative species, with their outer membrane rich in lipopolysaccharides (LPS), generally exhibit greater resistance (Bouftila et al., 2024[9]). Key indicators of membrane permeability disruption include potassium, protein, and nucleic acid leakage, as well as changes in membrane potential (Kashyap, 2024[35]). Phenolic-rich EOs, such as those containing carvacrol, eugenol, and thymol, are particularly effective in destabilizing bacterial membranes, leading to ion flux and loss of viability (Yap et al. 2021[65]). Disruption of membrane integrity triggers severe homeostatic imbalance, resulting in calcium toxicity, proteolysis activation, osmotic stress, and oxidative damage, ultimately leading to cell death. The efflux of potassium alters cellular conductivity and promotes water loss, causing cell shrinkage and apoptosis (Kashyap, 2024[35]). Di Pasqua et al. (2007[21]) further demonstrated that EO constituents interfere directly with membrane lipid biosynthesis, inducing structural and compositional alterations in bacterial membranes. Imaging techniques like scanning and transmission electron microscopy confirm EO-induced membrane damage, revealing deformed, aggregated, or lysed bacterial cells. Additionally, zeta potential alterations suggest EO-induced surface charge modifications, particularly in Gram-negative bacteria, linked to LPS loss (Yap et al. 2021[65]).

### Disruption of Energy Production

In prokaryotes, ATP is produced through glycolysis, which takes place in both the cell wall and the cytosol. It is expected that the action of EOs on the cell membrane affect alterations in the balance between intracellular and extracellular ATP levels. Disruptions in the membrane caused by EO are believed to lead to ATP leakage, and a connection between intracellular and extracellular ATP concentrations has been observed (Faleiro, 2011[25]). Thus, EOs significantly disrupt microbial energy production by depleting ATP levels, inhibiting ATP synthesis, and impairing cellular respiration. ATP, the primary energy carrier, drops by 95% in *Klebsiella pneumoniae* treated with *Monarda didyma* EO, indicating both ATP leakage from membrane damage and reduced synthesis (Chen Y et al., 2023[14]). Beyond permeability disruption, EOs inhibit ATPase activity, preventing ATP generation while accelerating hydrolysis, leading to severe energy depletion. *M. didyma* EO treatment showed a dose-dependent decline in ATPase function, further impairing microbial metabolism (Chen Y et al., 2023[14]). Additionally, EOs interfere with the tricarboxylic acid cycle, reducing the activity of key enzymes such as citrate synthase (CS), isocitrate dehydrogenase (IDH), and α-ketoglutarate dehydrogenase (α-KGDH). This disruption weakens energy production, slows microbial growth, and promotes cell death (Chen Y et al., 2023[14]). Additionally, some EO compounds directly bind to ATP synthase, blocking its function through interactions with key amino acid residues, further amplifying ATP depletion (Issa et al., 2019[31]). Disruption of membrane fluidity leads to a reduction in organelle membrane potential, proton pump failure, and inhibition of H-ATPase, a critical enzyme in ATP production (Chen L et al., 2023[13]). 

As ATPase activity declines, dependent enzymatic pathways are also affected (Issa et al., 2019[31]). Protease and phospholipase activities are reduced due to ATPase degradation by EO components such as cinnamaldehyde (Chen L et al., 2023[13]). Fatty acid biosynthesis is impaired, inhibiting enzymes like acetyl-CoA carboxylase and phospholipid biosynthetic pathways, leading to biofilm disruption (Chen L et al., 2023[13]). Furthermore, EO exposure alters mitochondrial morphology and membrane potential in *Pseudomonas roqueforti*, severely damaging energy metabolism and ultimately triggering apoptosis (Ju et al., 2020[33]).

### Inhibition of Quorum Sensing and Biofilm Formation

Essential oils disrupt quorum sensing (QS) and biofilm formation by interfering with bacterial signaling pathways, membrane integrity, and metabolic activity (Faleiro, 2011[25]). Cinnamaldehyde and other EO components reduce membrane hydrophobicity, inhibiting bacterial adhesion and self-aggregation, which destabilizes biofilm structure (Chen L et al., 2023[13]). These compounds downregulate key QS genes, including *luxR* and *BcsA*, suppressing the synthesis of signaling molecules in *Listeria monocytogenes* (Liu et al., 2021[45]). Carvacrol and eugenol further inhibit QS by binding to regulatory proteins, reducing the production of virulence factors (Rathinam et al., 2017[57]). Eugenol inhibits protease, pyocyanin, pyranan biosynthesis, extracellular polysaccharide, and rhamnolipid and closely binds to the synthesis of carbonyl N-coa acylation regulatory protein (LasR) by *P. aeruginosa*, thereby leading to the inhibition of QS (Rathinam et al., 2017[57]). Additionally, EOs disrupt intracellular ATP levels, altering mitochondrial membrane potential and ultimately leading to bacterial apoptosis (Ju et al., 2020[33]). In *Klebsiella pneumoniae*, biofilm formation is significantly reduced by thymol-rich *M. didyma* EO, which downregulates biofilm-associated genes and limits exopolysaccharide production (Chen Y et al., 2023[14]). Similarly, Qian et al. (2019[54]) reported that eugenol, at MIC concentration, strongly inhibited biofilm formation in carbapenem-resistant *Klebsiella pneumoniae* (CRKP-12). This effect was confirmed by FESEM and CLSM imaging, which revealed compromised biofilm integrity. Moreover, qRT-PCR analysis showed that eugenol downregulated key biofilm-related genes such as pgaA, luxS, wbbM, and wzm, while mrkA was upregulated. CLSM images further revealed extensive cell membrane damage in biofilm-associated CRKP-12 cells treated with 2MIC eugenol for 4 hours, contrasting with intact cell membranes in untreated controls (Qian et al., 2019[54]).

### Oxidative Stress and Reactive Oxygen Species Generation.

Essential oils exert antimicrobial effects largely by inducing oxidative stress through the generation of reactive oxygen species (ROS), which disrupt bacterial homeostasis and cause cellular damage. ROS, such as superoxide anions (O₂⁻), hydrogen peroxide (H₂O₂), and hydroxyl radicals (OH• ), are highly reactive molecules that can damage essential macromolecules, including DNA, lipids, and proteins. Bowbe et al. (2023[10]) demonstrated that *R. officinalis* and *M. communis* EOs, both individually and in combination, significantly increased ROS production in *S. aureus*, with the strongest oxidative stress observed for a 1:1 mixture of *R. officinalis* and *M. communis*. This ROS overproduction, in a dose-dependent manner, aligns with prior findings that Chamomile EO triggers oxidative stress in *S. aureus*. Similarly, Kong et al. (2022[41]) reported that lavender EO induces ROS-mediated membrane disruption in *K. pneumoniae*, while cinnamon bark EO affects energy production and DNA repair. Bacteria counteract oxidative stress through antioxidant enzymes like catalase (CAT) and superoxide dismutase (SOD), which detoxify ROS. However, Bowbe et al. (2023[10]) found that EO-treated *S. aureus* cells exhibited reduced catalase activity, mirroring previous observations with phyto-compounds like *Leonurus cardiaca* extract and catechin. While some studies indicate that EOs may enhance bacterial antioxidant responses initially, prolonged exposure overwhelms defense mechanisms, leading to oxidative stress-induced cell death. The dual role of certain compounds as both antioxidants and prooxidants further underscores the complex interactions between EOs and bacterial redox balance.

### Enzymatic Inhibition and Metabolic Pathway Disruption

Eugenol alters the bacterial fatty acid profile, compromising cytoplasmic membranes and disrupting enzymes like ATPase, amylase, histidine carboxylase, and proteases (Swamy et al., 2016[61]). Cinnamaldehyde inhibits ATPase and damages the outer membrane, while carvacrol impairs protein folding (DnaK, GroEL) and inhibits flagellin synthesis, reducing bacterial motility (Swamy et al., 2016[61]). Thymol significantly reduces oxidative metabolism by inhibiting TCA cycle and PPP pathway enzymes, with G6PDH, CS, IDH, and α-KGDH activity decreasing by 10% to 100% depending on conditions (Chen Y et al., 2023[14]). ATPase inhibition further impairs proteases, phospholipases, and fatty acid synthases (fasI, fasH, fasF), alongside glycerophospholipid biosynthesis enzymes (plsX, plsY, plsC, cdsA, pgsA, cls, mprF), aggravating bacterial biofilm damage (Chen Y et al., 2023[14]).

### Interaction with Genetic Material

Essential oils, particularly thymol and eugenol, demonstrate antimicrobial activity by directly interacting with bacterial genetic material. These compounds can bind to DNA and RNA, causing strand breakage or inhibiting replication and transcription, ultimately halting bacterial growth (Faleiro, 2011[25]). Thymol has been shown to bind directly to the genomic DNA of S. aureus, inducing DNA aggregation and altering its structure, which disrupts gene expression and protein synthesis (Wang et al., 2016[63]). This interaction, alongside thymol's membrane-disrupting properties, enhances its antibacterial efficacy. Additionally, thymol's binding to DNA's minor groove may induce secondary structure changes, further impairing bacterial function. The genotoxicity and antimutagenic potential of essential oils have been evaluated using assays like the Ames test and the SOS-Chromotest, which assess DNA damage and repair mechanisms (Wang et al., 2016[63]). 

### Epigenetic Modulation by Essential Oils

Emerging evidence suggests that essential oils can exert part of their antibacterial activity through epigenetic mechanisms. Recent studies have demonstrated that sub-inhibitory concentrations of EOs, particularly from *Origanum vulgare*, *Clinopodium nepeta*, and *Foeniculum vulgare*, can induce significant changes in the methylation profiles of bacterial DNA (D'Aquila et al., 2022[15]; D'Aquila et al., 2023[17]). Specifically, treatments with EOs were shown to modify the levels of 5-methylcytosine and N6-methyladenosine residues in *Escherichia coli* JM109 parental and antibiotic-resistant strains. These modifications can result in the disruption of critical processes such as quorum sensing, biofilm formation, and virulence expression. In most cases, EO exposure led to increased methylation levels at both cytosine and adenine residues, although variability was noted depending on the EO type and bacterial resistance profile (D'Aquila et al., 2022[15]). Such epigenetic changes contribute to the antimicrobial activity of EOs through modulation of bacterial gene regulation pathways. 

## 4. Major Foodborne Pathogens and their Interaction with Essential Oils

Foodborne diseases remain a critical public health challenge, with several pathogens posing significant threats. The 2023 European Union One Health Zoonoses Report (EFSA, 2024[24]) identified *Salmonella* and *Campylobacter* as the most frequently reported zoonotic diseases, with both showing an increase in cases compared to 2022. *Escherichia coli* (specifically Shiga toxin-producing *E. coli* or STEC) and *Listeria monocytogenes* were also highlighted as major concerns. Given their prominence in the EFSA report, this review will focus on these four major foodborne pathogens and their interactions with essential oils.

### 4.1. Salmonella spp.

*Salmonella* spp. continue to pose a major public health threat, being responsible for a significant number of foodborne illness outbreaks. In 2022, the EU reported over 65,000 cases of salmonellosis, with contaminated eggs, meat, and salad products being primary sources (EFSA, 2024[24]). Essential oils (EOs), especially those rich in phenolic compounds, have demonstrated potent antimicrobial properties against these pathogens. For instance, Galgano et al. (2023[28]) investigated the antibacterial and biofilm inhibition activity of *Thymus vulgaris* L. essential oil (TEO) against *Salmonella* on poultry litter. The study revealed that TEO, rich in thymol (47%) and p-cymene (19.6%), exhibited significant antimicrobial activity, with minimum inhibitory concentrations and effectively inhibited biofilm production in *Salmonella* isolates, suggesting its potential as a natural antimicrobial agent in controlling *Salmonella* infections. Similarly, Rochín-Medina et al. (2023[58]) evaluated the antimicrobial activity of oregano (*Origanum vulgare*) and thyme (*Thymus vulgaris*) essential oils against clinically and environmentally isolated *Salmonella* serotypes, including *S. Saintpaul, S. Oranienburg*, and *S. Infantis*. The study found that oregano essential oil had the highest antimicrobial activity, with MIC values of 0.1 μL/mL for all serotypes. Molecular docking analysis suggested that compounds like thymol and carvacrol interact optimally with microbial enzymes, contributing to their antimicrobial efficacy. Cinnamon essential oil (CEO) has been shown to exert significant antimicrobial effects against *S. Enteritidis*. Zhang et al. (2022[67]) study have demonstrated that CEO induces oxidative stress within bacterial cells, leading to an accumulation of reactive oxygen species (ROS) and disruption of the antioxidant enzyme system, including superoxide dismutase (SOD), catalase (CAT), and peroxidase (POD). This oxidative damage results in increased malondialdehyde (MDA) content and protein carbonylation, impairing bacterial growth. Additionally, CEO affects the expression of outer membrane protein genes (OmpF, OmpA, and OmpX), indicating disturbances in cell membrane function. Furthermore, CEO has been observed to alter the secondary structure of bacterial proteins, as evidenced by shifts in amide I and II bands in Fourier-transform infrared (FT-IR) spectra, and to reduce the fluorescence intensity of membrane proteins, suggesting conformational changes. CEO also inhibits intracellular β-galactosidase activity, further disrupting bacterial metabolism (Zhang et al., 2022[67]).

### 4.2. Campylobacter spp. 

According to Ramić et al. (2022[56]), lavandin essential oils (EOs) exhibit potent antimicrobial activity against *Campylobacter jejuni*, with minimum inhibitory concentrations of 0.25 mg/mL. The mechanism of action of these EOs is primarily attributed to their rich composition in bioactive terpenes, particularly linalool and linalyl acetate. Linalool, the dominant terpene alcohol in lavandin EOs, disrupts bacterial cell membranes by increasing membrane permeability, leading to ion leakage, loss of intracellular content, and ultimately bacterial cell death. The hydrophobic nature of these EO constituents also enables them to integrate into the lipid bilayer of bacterial membranes, further compromising membrane integrity. While lavandin EOs were less effective in modulating Campylobacter intercellular signaling compared to ethanolic extracts, they significantly inhibited bacterial adhesion and biofilm formation at subinhibitory concentrations (0.25 × MIC). This anti-adhesive effect likely results from EO interference with surface proteins or extracellular polymeric substances essential for bacterial attachment (Ramić et al., 2022[56]). In a separate study by Mutlu-Ingok and Karbancioglu-Guler (2017[48]), *Campylobacter jejuni and* *Campylobacter coli* were exposed to cardamom, cumin, and dill weed EOs. The results showed that these EOs compromised the bacterial cell membrane integrity. Specifically, cardamom EO caused a 100% increase in membrane permeability in *C. jejuni*, suggesting a complete release of electrolytes from the cell. The increase in relative electrical conductivity was accompanied by a substantial release of cellular contents, such as proteins and nucleic acids. Additionally, extracellular ATP concentrations were significantly elevated upon EO treatment indicating that the EOs induced a loss of ATP from the intracellular to the extracellular medium. The findings of Mutlu-Ingok and Karbancioglu-Guler (2017[48]) demonstrate that the antimicrobial action of the EOs is primarily due to their ability to disrupt the cytoplasmic membrane, leading to leakage of cellular materials and loss of intracellular ATP, ultimately resulting in cell lysis and bacterial death.

### 4.3. Escherichia coli (particularly E. coli O157:H7 and other Shiga toxin-producing strains)

*E. coli *O157:H7 and other Shiga toxin-producing strains (STEC) have a significant impact on human health, with a 30% increase in infections from 2022 to 2023 (EFSA, 2024[24]). Kim et al. (2016[40]) demonstrated that bay, clove, pimento berry essential oils, and particularly eugenol effectively inhibit Shiga toxin-producing *Escherichia coli* (EHEC), including strains like *E. coli* O157:H7 biofilm formation by down-regulating genes involved in biofilm production and virulence. According to these authors, Transcriptional analysis revealed that eugenol down-regulated key genes involved in biofilm formation and virulence, such as curli and type I fimbriae genes, along with LEE-encoded toxin genes (espD, escJ, escR, and tir). Additionally, biocompatible poly(lactic-co-glycolic acid) coatings containing eugenol or clove essential oil effectively inhibited biofilm formation on solid surfaces, reducing the virulence of EHEC without harming laboratory *E. coli* biofilms (Kim et al., 2016[40]). Al-Nabulsi et al. (2020[2]) showed that thyme and cinnamon EOs significantly reduce *E. coli* O157:H7 viability in tahini, a Mediterranean food, across various temperatures. According to the authors, the addition of thyme or cinnamon EOs at concentrations of ≤ 2.0% effectively inhibits *E. coli* O157:H7 growth, especially when tahini or tahini-based products are stored over a wide temperature range (10 to 37 °C). Oussalah et al. (2006[50]) conducted a study to investigate the mechanism of action of Spanish oregano, Chinese cinnamon, and savory essential oils against *E. coli* O157:H7. The study found that all three essential oils were able to damage the cell membrane of *E. coli* O157:H7, leading to the leakage of ions and other cellular contents. The essential oils were also able to acidify the cytoplasm of the bacteria and disrupt its membrane potential. Additionally, the essential oils were able to denature proteins and disrupt membrane structure.

### 4.4. Listeria monocytogenes

The mechanism of action of EOs against *Listeria monocytogenes* involves multiple antibacterial strategies, targeting both planktonic cells and biofilm structures. The phenolic compounds carvacrol and thymol, key components in oregano and thyme EOs, play a crucial role in their antimicrobial activity. These compounds integrate into the bacterial cell membrane, disrupting its lipid bilayer structure. This disturbance compromises membrane integrity, increasing permeability and causing leakage of cellular contents (Vidaković Knežević et al., 2022[62]). Additionally, carvacrol and thymol act as proton exchangers, reducing the proton gradient across the cytoplasmic membrane, which leads to the inhibition of the respiratory chain, disruption of electron transport, and alteration of intracellular pH. Consequently, this results in ATP depletion, loss of essential cellular components, and eventual cell death. Moreover, these EOs exhibit strong antibiofilm properties, effectively reducing *L. monocytogenes* biofilm biomass by 32.61% to 78.62% (Vidaković Knežević et al., 2022[62]). This is particularly important as mature biofilms provide significant resistance to antimicrobial agents due to their extracellular polysaccharide matrix and the activation of protective bacterial genes. In addition, *Chrysanthemum* essential oil (CHEO) has demonstrated potent antibacterial activity against *L. monocytogenes* by significantly increasing membrane permeability and disrupting the bacterial cell membrane, resulting in the leakage of intracellular substances. Lin et al. (2019[44]) reported that CHEO treatment reduced the quantification of DNA, protein, and ATP in *L. monocytogenes* by 73.58%, 70.01%, and 84.63%, respectively, as compared to the control group. CHEO-treated cells exhibited an increased surface Zeta potential, indicating cell agglutination and accelerated bacterial death. CHEO exposure also elevated the electrical conductivity and adsorption rate in *L. monocytogenes*, suggesting electrolyte leakage due to membrane damage. CHEO also inhibited the respiratory metabolism by suppressing key enzymes in the Embden-Meyerhof-Parnas (EMP) pathway, including hexokinase, phosphofructokinase, and pyruvate kinase (Lin et al., 2019[44]). Similarly, basil EO (BEO) exerts antibacterial effects by increasing cell membrane permeability and inducing intracellular leakage of proteins and DNA. Using spectroscopy technology and molecular docking prediction, Li and co-workers (2022) have identified BEO's multi-target antibacterial mechanism. Linalool, a major component of BEO, interacts with the hydrocarbyl chain of the phospholipid tail, affecting cell membrane structure and function. Additionally, molecular docking results revealed that α-bergamotene interacts with key enzyme amino acid residues, inhibiting bacterial respiratory metabolism by targeting essential enzymes such as phosphofructokinase (PFK) and pyruvate kinase (PK). This disruption in the Embden-Meyerhof-Parnas (EMP) pathway impairs bacterial energy production, further enhancing BEO's antibacterial effect (Li et al., 2022[43]).

### 4.5. Essential oils application methods

As discussed in this paper, using essential oils (EOs) as an eco-friendly preservative is recommended to extend food shelf life. Additionally, EOs can serve as valuable flavoring and aromatizing agents. For example, citrus EO is commonly used to flavor tea, liqueurs, sweets, and candied fruits (Di Bella et al., 2006[20]). Despite their promising bioactivity, essential oils face significant hurdles in food applications, including limited solubility, strong aromas that alter sensory properties, and compositional variability. Beyond these technical barriers, their potential toxicity at high doses and the lack of harmonized regulatory frameworks raise safety concerns, prompting the need for alternative delivery methods. Accordingly, the direct addition of EOs to food-the most straightforward approach-struggles with volatility, poor solubility, and sensory changes that often outweigh their antimicrobial benefits. To overcome these limitations, emulsification techniques, including nano- and micro-emulsions, are increasingly employed to improve EO dispersion, stability, and bioavailability in food matrices (Figure 3(Fig. 3)). Table 3(Tab. 3) (References in Table 3: Ahmed et al., 2021[1]; Blanco-Lizarazo et al., 2019[8]; Chaichi et al., 2021[12]; Dai et al., 2020[16]; Ed-Dra et al., 2018[23]; He et al., 2021[30]; Jayari et al., 2019[32]; Kang et al., 2019[34]; Kazemeini et al., 2019[37], 2021[36]; Khaleque et al., 2016[38]; Khezri et al., 2021[39]; Lin et al., 2019[44]; Öztürk et al., 2021[51]; Passafiume et al., 2022[52]; Radunz et al., 2020[55]; Yuan et al., 2019[66]) provides a compilation of such studies, detailing the specific pathogens targeted, food products analyzed, application methods, concentrations used, and observed outcomes.

***The direct application*** of essential oils involves their straightforward incorporation into food products to harness their potent antibacterial properties (Angane et al., 2021[4]). This method is both simple and cost-effective, providing immediate antimicrobial action against a broad spectrum of foodborne pathogens (Falleh et al., 2020[27]). Essential oils such as oregano (rich in carvacrol), clove (containing eugenol), and thyme (high in thymol) are frequently applied directly to fresh produce, meats, and dairy products, where they effectively inhibit spoilage microorganisms like *Listeria monocytogenes*, *Salmonella* spp., and *Escherichia coli.* In a study on minced pork meat stored at 4°C, oregano and thyme essential oils significantly reduced *L. monocytogenes* populations. When applied at concentrations of 0.36 µL/g and 0.72 µL/g, these oils achieved reductions of 0.51 log10 CFU/g and 0.29 log10 CFU/g, respectively, by the fourth day of storage (Vidaković Knežević et al., 2022[62]). Piper nigrum essential oil (PNEO) demonstrated notable antibacterial activity in red deer meat. After 14 days of storage at 5°C, samples treated with 1% PNEO exhibited a substantial 64% reduction in Total Viable Count compared to untreated samples (Vidaković Knežević et al., 2022[62]). In addition to meat preservation, the direct application of *Thymus capitatus* essential oil has shown remarkable benefits in improving milk quality and extending its shelf life. According to Ben Jemaa et al. (2018[6]), incorporating 1 mg/L of this EO into milk, particularly when combined with pasteurization, effectively inhibited bacterial growth and delayed milk spoilage. The EO's potent antimicrobial properties suppressed contaminant bacterial growth for up to 48 hours post-incubation, ensuring improved microbial stability. Moreover, *T. capitatus* EO reduced peroxide formation, thereby slowing milk fat oxidation and preserving freshness (Ben Jemaa et al., 2018[6]). Despite its advantages, the direct application of essential oils presents challenges such as strong flavors and aromas, poor solubility, volatility, susceptibility to degradation, and sensitivity to environmental factors like light, temperature, and pH (Falleh et al., 2021[26]; Ben Jemaa et al., 2017[6]). Ensuring uniform distribution and stability remains challenging, particularly in aqueous food systems. Due to their hydrophobic nature, essential oils may exhibit poor solubility, potentially resulting in uneven dispersion and reduced antimicrobial effectiveness (Falleh et al., 2020[27]).

The encapsulation techniques protect EOs from degradation, mask their strong flavors, and facilitate their homogeneous distribution in food matrices (Dghais et al., 2023[18]; Falleh et al., 2021[26]). Low concentrations of EOs could be incorporated into active packaging systems then added to food packaging films. These active packaging systems gradually release the volatile antimicrobial compounds onto the food's surface, where microbial contamination typically occurs, thus providing effective protection without compromising the food's acceptability (Casalini and Giacinti Baschetti, 2023[11]; Hassoun & Çoban, 2017[29]). In a similar context, Alonzo et al. (2024[3]) highlighted that the application of edible coatings enriched with cinnamon EO has shown potential for preserving a variety of food products. Casalini and Giacinti Baschetti (2023[11]) highlighted the growing interest in incorporating essential oils into sustainable food packaging systems. Their review focuses on the use of EOs in antimicrobial food packaging, emphasizing their potential to not only extend product shelf life but also enhance the functionality of packaging materials. The integration of EOs with biopolymers such as nanocellulose and chitosan is discussed as a promising approach for creating natural active packaging.

The antimicrobial effectiveness of EO-infused packaging systems showed positive effects on material properties such as tensile strength and water vapor transmission rate (Casalini and Giacinti Baschetti, 2023[11]). Another confirmed strategy to encapsulate EOs is their encapsulation into micro- or nanoemulsions. Indeed, nanoemulsions of thyme essential oil significantly reduced bacterial growth in contaminated milk, achieving a 34.7% reduction in bacterial counts compared to the use of free essential oil (Ben Jemaa et al., 2017[6]). Similarly, nanoencapsulation of cinnamon and curcuma essential oils (EOs) enhanced their antimicrobial activity against foodborne pathogens such as *Escherichia coli* and *Staphylococcus aureus*. The antimicrobial activity of free cinnamon EO showed an increase of 14.3% when encapsulated as a nanoemulsion, highlighting the improved efficacy of the nanoencapsulated form (Dghais et al., 2023[18]). Moreover, nanoencapsulation improved the oxidative stability of minced beef meat, reducing lipid oxidation and metmyoglobin formation, thereby extending shelf life (Dghais et al., 2023[18]). Actually, the small droplet size of nanoemulsions (e.g., 89 nm for cinnamon EO) increases the active surface area, enhancing their interaction with microbial cells and leading to faster and more pronounced antimicrobial effects (Falleh et al., 2021[26]). 

#### Controlled Release of EOs in Packaging.

EOs have demonstrated exceptional antimicrobial efficacy and are widely recognized as safe for oral consumption, with several classified as GRAS by the US-FDA (Anis et al., 2020[5]). To address their limitations, EO-containing antimicrobial packaging (EO-AP) systems have been designed, where EOs are entrapped within polymeric films. These films contain micropores that facilitate the controlled diffusion of EOs from the polymeric matrix to the food surface. This diffusion mechanism is crucial, as the sustained release of EOs ensures prolonged antimicrobial protection. EO-AP systems function either as reservoir systems, where EOs diffuse freely within the polymeric matrix, or as monolithic systems, where diffusion occurs unilaterally. In the former, if EOs are miscible with the food material, rapid diffusion may cause EO depletion below the minimum inhibitory concentration (MIC), compromising the antimicrobial effect. Conversely, in monolithic systems, EOs that are immiscible with food maintain stable diffusion rates, ensuring consistent EO levels throughout the storage period. Thus, designing an EO-AP system with an optimized release profile is critical to balancing effective microbial control with prolonged shelf life. The diffusion kinetics of EOs must be carefully calibrated to maintain EO concentrations above the MIC threshold. An excessively rapid diffusion may initially exceed the MIC but will lead to premature EO depletion, shortening the product's shelf life (Anis et al., 2020[5]). Conversely, diffusion that is too slow may result in EO concentrations insufficient to inhibit microbial proliferation. Moreover, understanding microbial growth kinetics is equally important, as a release rate slower than microbial proliferation would render the EO-AP system ineffective. Consequently, achieving an equilibrium between EO release and microbial growth dynamics is pivotal for ensuring sustained antimicrobial protection and extending food shelf life (Anis et al., 2020[5]).

#### Combined and Synergistic Effects of Essential Oil Components and preservative

Essential oils contain complex mixtures of bioactive compounds, and their interactions with other antimicrobials can lead to enhanced efficacy at lower concentrations (Falleh et al., 2020[27]). This reduces potential sensory, solubility, diffusion issues while maintaining strong antibacterial activity. Combinations of EOs can cause synergistic, additive, or antagonistic effects depending on the composition and concentration of the components. The synergistic effect occurs when the antimicrobial activity of the antimicrobial mixture is greater than the sum of the effects of the individual components. There is an additive effect if the antimicrobial activity is equal to the sum of the effects of the individual components, and there is an antagonistic effect if the antimicrobial activity is less than the sum of the effects of the individual components. Combined and synergistic effects of essential oil (EO) components have been increasingly recognized as a promising strategy for enhancing their preservative efficacy in food systems. For instance, the synergistic interaction between cinnamaldehyde and eugenol, major components of cinnamon and clove EOs, respectively, has been shown to significantly enhance antimicrobial activity against foodborne pathogens like *Staphylococcus aureus* (Falleh et al., 2020[27]). This synergy can be attributed to the complementary mechanisms of action, where cinnamaldehyde disrupts microbial ATPase enzymes and cell membranes, while eugenol permeabilizes bacterial membranes and binds to proteins. Additionally, the formulation of EO mixtures using experimental designs allows for the optimization of EO combinations to achieve maximum antimicrobial efficacy. For example, a mixture of 58.8% cinnamon, 34.4% clove, and 6.8% lavender EOs was found to be highly effective in reducing *S. aureus* contamination in milk (Falleh et al., 2020[27]). Another technique to reduce EO doses in food without compromising their activity is the use of their synergistic interaction with other antimicrobial agents. With this respect, Dhifi et al. (2016[19]) reported that the combinations of 1,8-cineole with aromadendrene exhibited an important protective effect against the methicillin-resistant *Staphylococcus aureus* (MRSA), the vancomycin-resistant *enterococci* (VRE) and the pathogen *Enterococcus faecalis*. Another valid approach is also actually discussed is the combined treatment process. The combination of sous-vide packaging with coriander essential oil was found to effectively preserve the quality of trout and ensure pathogen control, demonstrating the potential of this method for enhancing food safety and quality in seafood (Öztürk et al., 2021[51]). Finally, in several cases, the substitution of EO with its major chemical component may preserve the same activity without the organoleptic disadvantages, as this change eschews some compounds that modify food sensorial properties (Angane et al., 2021[4]).

## 5. Conclusions and Recommendations for Future Work

This review underscores the remarkable antimicrobial potential of essential oils (EOs), emphasizing their diverse mechanisms of action against foodborne pathogens. EOs have demonstrated their ability to disrupt bacterial cell membranes, increase permeability, and induce leakage of intracellular components, ultimately leading to cell death. Moreover, EOs interfere with bacterial energy production by depleting ATP levels and disrupting vital metabolic pathways such as the tricarboxylic acid cycle. Their capacity to generate reactive oxygen species (ROS) further enhances their antimicrobial properties by causing oxidative damage to bacterial DNA, proteins, and lipids. Additionally, EOs' ability to inhibit quorum sensing and biofilm formation significantly reduces bacterial virulence and resistance, making them a promising alternative to synthetic preservatives. Despite these advantages, certain challenges hinder the widespread application of EOs in the food industry. Factors such as strong flavors, limited solubility, and environmental sensitivity can compromise their effectiveness. While encapsulation techniques and EO combinations were addressed in this paper, future research should focus on studying EO interactions with food matrices on a case-by-case basis to ensure optimal performance. Additionally, assessing their long-term stability and identifying the optimal EO concentration that achieves effective preservation without toxic effects are crucial for their successful integration into food preservation systems. By overcoming these challenges, EOs can emerge as sustainable and efficient natural preservatives that contribute to safer and healthier food products. 

## Declaration

### Funding

The authors would like to acknowledge the financial support provided by The Tunisian Ministry of Higher Education and Scientific research (LR15CBBC06).

### Acknowledgment

Special thanks are extended to Dr. Mariem Ben Jemaa for her valuable help in reviewing and providing constructive feedback on the manuscript, and to Dr. Nahla Ben Hmida for her precious contribution to the development of the visual illustration of Figure 1(Fig. 1).

### Conflicts of Interest

The authors declare no conflict of interest.

### Using artificial intelligence (AI) 

**Figure 2:**
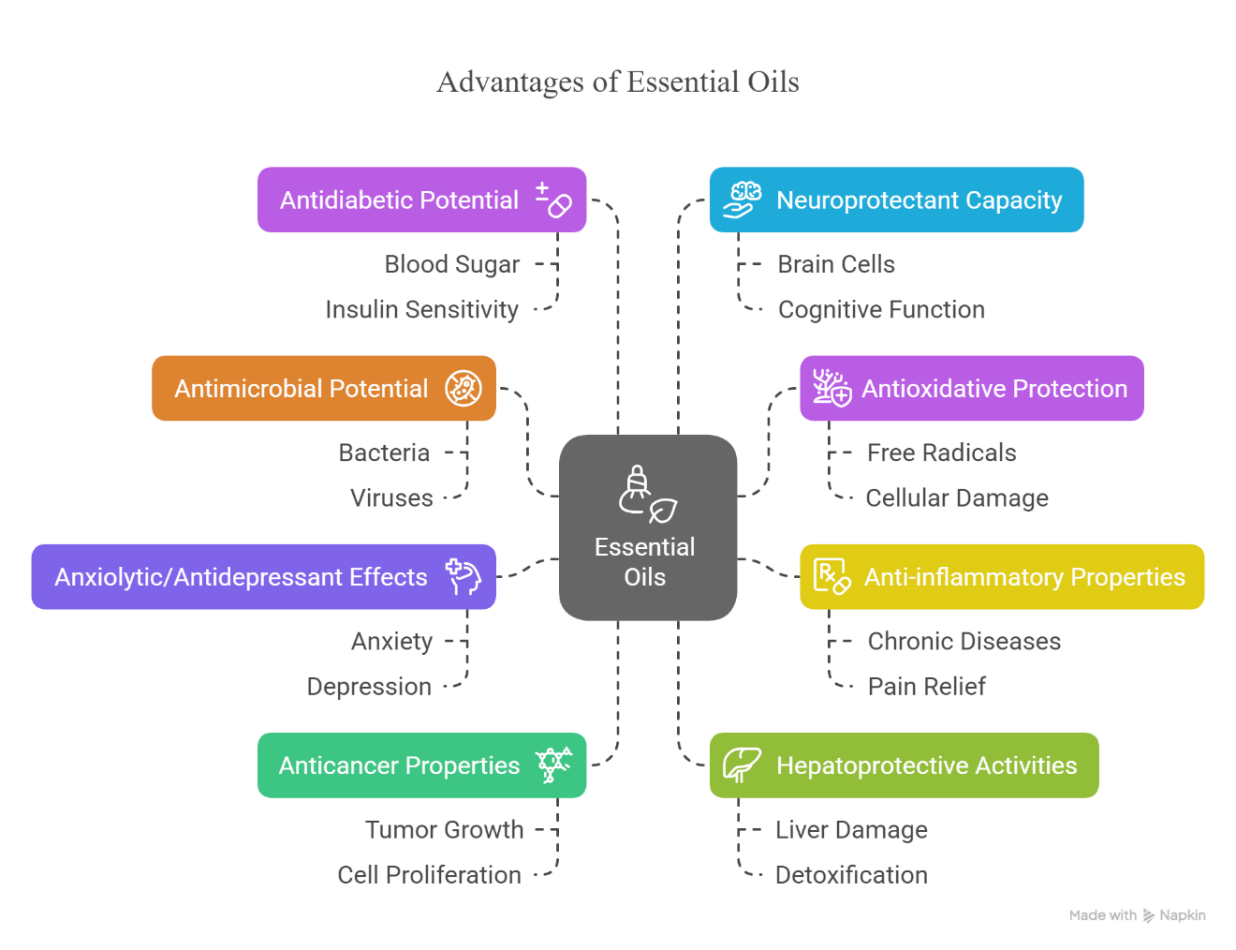
Main biological activities of common essential oils, including antimicrobial, anti-oxidative, anti-inflammatory, anxiolytic, antidepressant, anticancer, hepatoprotective, anti-diabetic, and neuroprotective properties.

**Figure 3:**
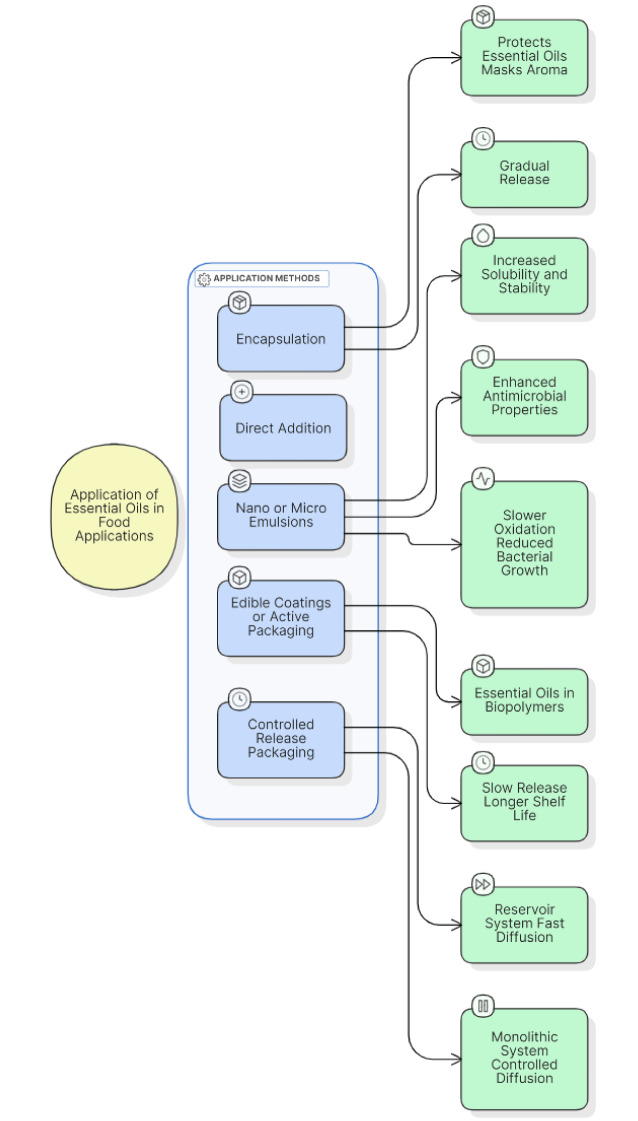
Flow Chart of the main application methods of Essential Oils in food.

The authors acknowledge the use of AI tools for language refinement, writing assistance, and support in the simple presentation of Figures 2(Fig. 2) and 3(Fig. 3).

## Figures and Tables

**Table 1:**
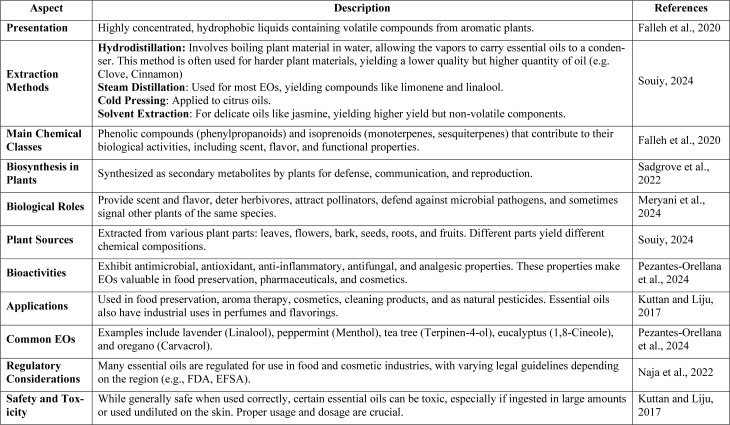
Overview of essential oils (EOs), including their definition, extraction methods, main chemical classes, biological functions, biosynthesis, roles in plant defence and industrial applications.

**Table 2:**
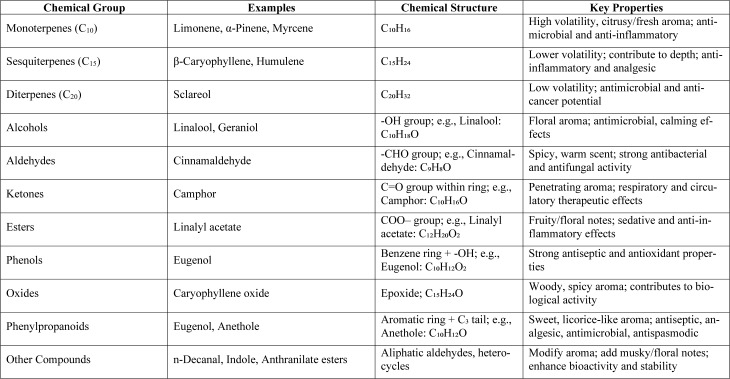
Chemical composition of essential oils, examples and key classes of molecules (Sadgrove et al., 2022, Falleh et al., 2020).

**Table 3:**
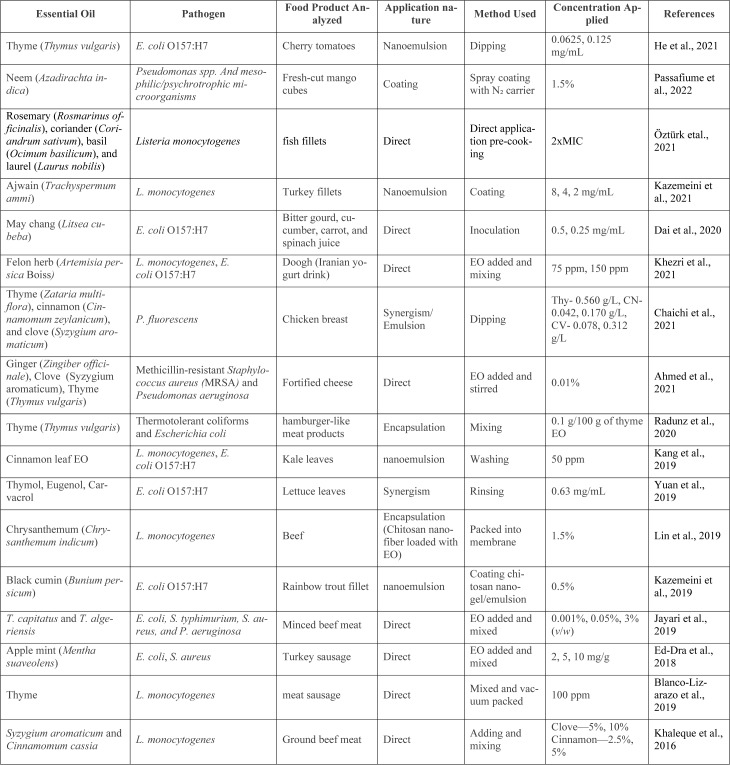
Table 3: Compilation of studies evaluating the antimicrobial activity of essential oils against specific pathogens in diverse food products, detailing application methods, concentrations, and outcomes.
